# Altered Cytokine Gene Expression in Peripheral Blood Monocytes across the Menstrual Cycle in Primary Dysmenorrhea: A Case-Control Study

**DOI:** 10.1371/journal.pone.0055200

**Published:** 2013-02-04

**Authors:** Hongyue Ma, Min Hong, Jinao Duan, Pei Liu, Xinsheng Fan, Erxin Shang, Shulan Su, Jianming Guo, Dawei Qian, Yuping Tang

**Affiliations:** Jiangsu Key Laboratory for TCM Formulae Research, Nanjing University of Chinese Medicine, Nanjing, China; Institut Jacques Monod, France

## Abstract

Primary dysmenorrhea is one of the most common gynecological complaints in young women, but potential peripheral immunologic features underlying this condition remain undefined. In this paper, we compared 84 common cytokine gene expression profiles of peripheral blood mononuclear cells (PBMCs) from six primary dysmenorrheic young women and three unaffected controls on the seventh day before (secretory phase), and the first (menstrual phase) and the fifth (regenerative phase) days of menstruation, using a real-time PCR array assay combined with pattern recognition and gene function annotation methods. Comparisons between dysmenorrhea and normal control groups identified 11 (nine increased and two decreased), 14 (five increased and nine decreased), and 15 (seven increased and eight decreased) genes with ≥2-fold difference in expression (*P*<0.05) in the three phases of menstruation, respectively. In the menstrual phase, genes encoding pro-inflammatory cytokines (IL1B, TNF, IL6, and IL8) were up-regulated, and genes encoding TGF-β superfamily members (BMP4, BMP6, GDF5, GDF11, LEFTY2, NODAL, and MSTN) were down-regulated. Functional annotation revealed an excessive inflammatory response and insufficient TGF-β superfamily member signals with anti-inflammatory consequences, which may directly contribute to menstrual pain. In the secretory and regenerative phases, increased expression of pro-inflammatory cytokines and decreased expression of growth factors were also observed. These factors may be involved in the regulation of decidualization, endometrium breakdown and repair, and indirectly exacerbate primary dysmenorrhea. This first study of cytokine gene expression profiles in PBMCs from young primary dysmenorrheic women demonstrates a shift in the balance between expression patterns of pro-inflammatory cytokines and TGF-β superfamily members across the whole menstrual cycle, underlying the peripheral immunologic features of primary dysmenorrhea.

## Introduction

Primary dysmenorrhea is one of the most common gynecological complaints and is characterized by pain, cramping, and backache occurring during menses in young women [1]. It affects up to 50% of women at some point in their reproductive life resulting in a significant socio-economic impact [2]. It is thought to be caused by an exaggerated response to physiological processes at the time of menstruation and there is some evidence that women with primary dysmenorrhoea experience uterine hypercontractility in the perimenstrual phase [3], [4]. During contractions, uterine blood flow is compromised, leading to relative tissue ischemia and pain. Analysis of peripheral blood from dysmenorrhoeic women revealed excessive synthesis and concentrations of oxytocin (OT), PGF2α, vasopressin (VAP) and IL-6 [5], [6], [7], [8]. On the first day of menstruation, significantly higher plasma vasopressin and PGF2α metabolite levels were found in severe primary dysmenorrhea women [7]. The plasma oxytocin and IL-6 concentrations were also markedly higher in the dysmenorrheic patients than in the healthy volunteers at menstruation [8]. These mediators could increase uterine contractility [9] and have important roles in the pathophysiology of primary dysmenorrhea.

Primary dysmenorrhea may be associated with dysregulation of normal menstruation. Menstruation is a response to the withdrawal of progesterone and depends on the complex interactions between ovarian hormones and the immune system [9]. A variety of immune factors in the endometrium contribute to decidualization, menstruation and subsequent repair [10]. Decidualization at the end of the secretory phase is a differentiation process crucial to prepare the endometrium for embryo implantation or menstruation. Many cytokines have been identified which could enhance or inhibit decidualization, including IL-1, TNFα, LEFTY, bone morphogenetic proteins (BMPs) and GSF2 [9]. In the absence of conception, falling circulating progesterone levels in the late secretory phase of the cycle trigger leukocyte influx and activation, followed by production of protease (MMPs), which cause sloughing of the decidualized superficial endometrial layer and menstruation. Meanwhile, self-limiting menstrual inflammation has a direct influence on post-menstrual repair. For example, hypoxic conditions allow translocation of HIF-1 to the nucleus and increased transcription of genes with hypoxic response elements, including those involved in tissue remodeling and angiogenesis (e.g. *VEGF, CTGF, Ang-2, CXCL8*) [9]. Although significant progress has been made in the mechanism of menstruation, the etiology of primary dysmenorrhea has not been clearly elucidated.

Gene expression analyses have been widely used to study human diseases. The studies compare gene expression profiles in disease and non-disease states to identify disease biomarkers and gain insights into pathophysiological processes. But the gene expression profiles in primary dysmenorrhea have not been reported. In this study, the real-time PCR array was used to determine 84 common cytokine gene expressions of peripheral blood mononuclear cells (PBMCs) from young women with primary dysmenorrhea and unaffected controls. We demonstrated that cytokine expression in PMBCs is dysregulated throughout the menstrual cycle in women suffering from dysmenorrhea and determined the underlying biological mechanisms involved.

## Materials and Methods

### Participants

Ethical approval for this study was obtained from Ethics Committee of Nanjing University of Chinese Medicine and Jiangsu Province Hospital on Integration of Chinese and Western Medicine, and informed written consent was obtained from all participants before sample collection. The experimental procedures were carried out in accordance with the Declaration of Helsinki and related ethical regulations of our university.

We recruited and selected our participants from Nanjing University of Chinese Medicine. Six volunteers aged 20–24 years were enrolled. They had cycles lasting 21–35 days with the actual menses periods lasting three to seven days, and they experienced at least 4 consecutive painful periods in the past six months with the pain starting one day before or on the day of onset of bleeding. The control group included three young women without any pain during menstruation. Moreover, these young women were not married, not taking oral contraceptive pills or other drugs, and they had no gastrointestinal, gynaecological or autoimmune diseases, or gynaecological surgery.

### Sample Collection and Processing

Blood (5 ml) was collected from six primary dysmenorrheic and three control women. Samples were divided into six groups: dysmenorrhea group on the seventh day before menstruation (DS), dysmenorrhea group on the first day of menstruation (DM), dysmenorrhea group on the fifth day of menstruation (DP), unaffected group on the seventh day before menstruation (NS), unaffected group on the first day of menstruation (NM), and unaffected group on the fifth day of menstruation (NP). The three blood sample collection time points were in the secretory, menstrual and proliferative phases of the menstrual cycle, respectively. The blood was collected into tubes containing sodium heparin at Nanjing University of Chinese Medicine. PBMCs were isolated from whole blood by Ficoll density gradient centrifugation using standard methods [11]. Isolated cells were counted and tested for viability by trypan blue exclusion prior to culture. Blood plasma was used for hormone assays to confirm the cycle stage; hormones measured were pregnendione (P4), estradiol (E2), follicle-stimulating hormone (FSH), and luteinizing hormone (LH).

### RNA Extraction

Total RNA was isolated from PBMCs with Trizol Reagent, using the guanidinium thiocyanate phenol-chloroform method according to the manufacturer’s instructions. Contaminating DNA was removed from RNA preparations using DNase I. The yield and quality of total RNA was determined according to the ratio of spectrophotometric absorbance values at wavelengths of 260 and 280 nm. RNA quality was further determined by denaturing agarose gel electrophoresis.

### Cytokine Gene Expression by qRT-PCR

cDNA synthesis was performed using DNase-treated RNA and random decamer primers in a final volume of 13 µl. The samples were heated for 5 min at 65°C and then placed on ice for 5 min. The 13 µl sample was then mixed with 1 µl of 0.1 M DTT, 1 µl of RNase Inhibitor and 1 µl of SuperScript III RT. The reaction mixture was incubated for 10 min at room temperature and then at 50°C for 60 min and 70°C for 15 min in a thermocycler. The cDNA generated was used as a template for quantitative real-time PCR (QPCR) [12]. A mastermix was prepared containing 44.8 µl of water, 55 µl of RT^2^ SYBR Green/ROX PCR Master Mix, and 10.2 µl of cDNA. This mixture was added to 96 wells in an RT^2^ Profiler™ PCR Array, Human Common Cytokines (PAHS-012A; QIAGEN). This PCR array contained RT^2^ qPCR Primer Assays for a set of 84 cytokines, including *BMP1, BMP2, BMP3, BMP4, BMP5, BMP6, BMP7, BMP8B, CSF1, CSF2, FAM3B, FASLG, FIGF, GDF10, GDF11, GDF2, GDF3, GDF5, MSTN, GDF9, IFNA1, IFNA2, IFNA4, IFNA5, IFNA8, IFNB1, IFNG, IFNK, IL10, IL11, IL12A, IL12B, IL13, TXLNA, IL15, IL16, IL17A, IL17B, IL17C, IL25, IL18, IL19, IL1A, IL1B, IL1F10, IL1F5, IL1F6, IL1F7, IL1F8, IL1F9, IL2, IL20, IL21, IL22, IL24, IL3, IL4, IL5, IL6, IL7, IL8, IL9, INHA, INHBA, LEFTY2, LTA, LTB, NODAL, PDGFA, TGFA, TGFB1, TGFB2, TGFB3, TNF, TNFRSF11B, TNFSF10, TNFSF11, TNFSF12, TNFSF13, TNFSF13B, TNFSF14, TNFSF4, CD70* and *TNFSF8.* The housekeeping genes are *B2M, HPRT1, RPL13A, GAPDH* and *ACTB*. The PCR array also contained a negative control to test for contaminating DNA, three reverse transcription controls and three positive PCR controls. A total 27 PCR array plates were used. The standard cycling conditions were as recommended by the PCR array supplier. Data were collected at the end of the annealing step. Fold changes in gene expression between the affected and control groups were calculated using the ΔΔCt method in the PCR array data analysis template. A simple examination of Ct value consistency for the housekeeping genes indicated that the normalization method performed adequately. A similar evaluation of the built-in RNA controls provided the relative levels of genomic DNA contamination and inhibitors of either the reverse transcription or PCR.

### Multivariate Data Analysis

Gene expression data (2^−ΔCt^) were transferred to MassLynx V4.1 software (Waters Corp., Milford, USA) for principal component analysis (PCA), partial least-squares discriminant analysis (PLS-DA) and orthogonal projection to latent structures (OPLS) analysis. PCA is an unsupervised multivariate statistical approach used for variable reduction and separation into classes. To maximize class discrimination and biomarkers, the data were further analyzed using the OPLS-DA method. S-plots were calculated to visualize the relationship between covariance and correlation within the OPLS-DA results. Variables that had significant contributions to discrimination between groups were considered as potential biomarkers. In the score plot, the scores t[1] and t[2] are the two most important new indices in summarizing and separating the data. The plot of t[1] vs. t[2] shows a picture of the data. Each point in the plot corresponds to an observation. The groups are differentiated by color, to facilitate their visual separation.

### Gene Functional Annotation and Biological Network Building

The database for annotation, visualization and integrated discovery (DAVID, http://david.abcc.ncifcrf.gov/) is a Web-based application that provides a high-throughput, integrative gene functional annotation environment to extract biological themes behind large gene lists systematically. Briefly, a gene list was uploaded, and then text and pathway-mining tools (i.e. a functional annotation chart) were used to search for potential signaling pathways or biological processes [13].

Genes encoding factors interacting with pro-inflammatory cytokines and BMPs were analyzed by SciMiner (http://jdrf.neurology.med.umich.edu/SciMiner/), as were genes associated with menstrual events. SciMiner is a Web-based literature mining and functional analysis tool that identifies genes using context-specific analysis of MEDLINE abstracts and full texts. SciMiner accepts a free text query (PubMed Entrez search) or a list of PubMed identifiers as input. Ambiguous acronyms are resolved by a scoring scheme based on the co-occurrence of acronyms and corresponding description terms, which incorporates optional user-defined filters. Functional enrichment analyses are used to identify highly relevant targets (genes and proteins), pathways and protein-protein interaction networks by comparing identified targets from one search result with those from other searches or to the full HGNC [HUGO (Human Genome Organization) Gene Nomenclature Committee] gene set [14]. Genes mediating interaction of pro-inflammatory cytokines and BMPs involved in the PGF2αproduction pathway, were further selected from the SciMiner gene list by reading PubMed abstracts manually. The biological relationships of pro-inflammatory cytokines and BMPs were also extracted by ALIBABA (http://alibaba.informatik.hu-berlin.de/) [15]. Finally, all extracted information relating to interactions was inputted into Cytoscape (http://www.cytoscape.org/) to construct a biological network.

### Statistical Analysis

Results of gene expression analyses are expressed as mean±S.D. and were evaluated using the two-tailed unpaired Student’s t-test. *P*<0.05 was considered to be significant and *P*<0.01 very significant.

## Results

### Cytokine Gene Expression Pattern Recognition in PBMCs from Primary Dysmenorrheic and Control Women

**Figure 1:**
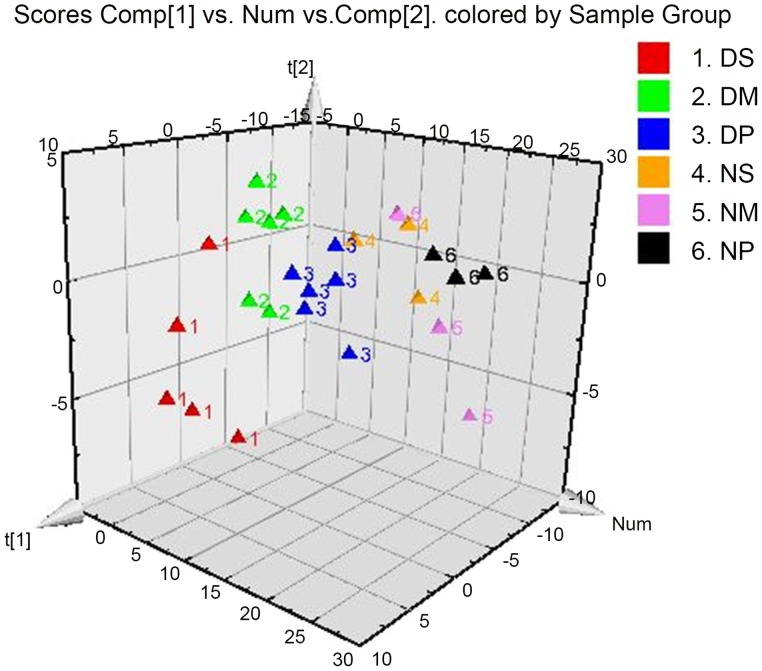
PLS-DA score plot (3D) of PBMC cytokine gene expression between controls and primary dysmenorrhea groups. DS, the secretory phase in the primary dysmenorrhea group (seventh day before menstruation); DM, the menstrual phase in the primary dysmenorrhea group (first day of menstruation); DP, the proliferative phase in the primary dysmenorrhea group (fifth day of menstruation); NS, NM, NP, the secretory, menstrual and proliferative phase, respectively, in unaffected controls.

In order to visualize the subtle similarities and differences among data sets across the whole menstrual cycle, multiple pattern recognition methods were employed to determine the cytokine gene expression phenotype in PBMCs. Here, PCA, PLS, and OPLS-DA were used to classify the expression profiles and identify differentiating genes. With PLS-DA, identification of discriminatory variables proceeds from an analysis of the PLS weights. In the PLS-DA score plots, each point represents an individual sample. Groupings and trends can be observed in all samples. The supervised PLS-DA analysis revealed substantial differences in the gene expression signature of the PBMCs from dysmenorrhea and control groups in the secretory, menstrual, and proliferative phases of menstruation (R^2^X [1] = 0.183, R^2^X [2] = 0.137, Figure 1). It can be concluded that cytokine gene expression profiles of PBMCs from dysmenorrheic women deviated from the normal states, and are more spatially dispersed, which may signify significant pathobiological changes.

**Figure 2:**
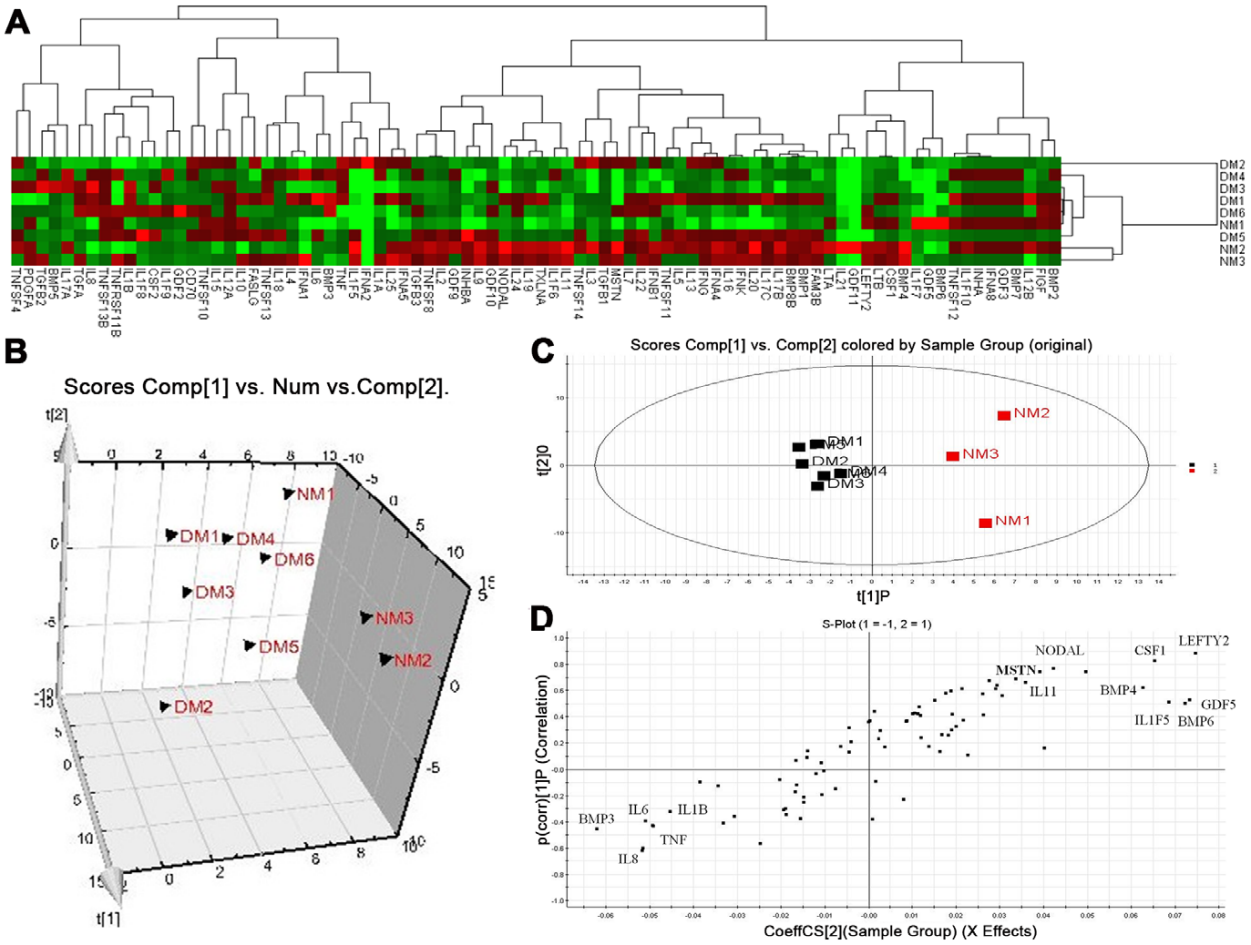
Multivariate analysis of cytokine gene expression profiles from controls (NM1-NM3) and dysmenorrheic (DM1–DM6) samples on the first day of menstruation. A) Heat map showing hierarchical clustering of individual arrays by gene expression. B) 3D PCA score plot showing separate clustering of expression profiles corresponding to DM vs. NM. C) OPLS-DA model results for DM vs NM group. D) S-plot of OPLS-DA model for DM vs. NM group.

Heat maps based on average fold-change (FC) in expression for each gene in the array were generated to visualize the level of correlation between unaffected control (NM) and dysmenorrhea samples (DM) on the first day of menstruation (Figure 2A). Data were subjected to PCA and OPLSA to distinguish clear clusters. The PCA model (unsupervised multivariate analysis method) provides an overview of all observations or samples in a data set and results are displayed as score plots, indicating the scatter of the samples. Similar genomic compositions are represented by clusters and compositionally different genomes are indicated when the sample scatter is dispersed. In Figure 2B, the PCA score plot clearly separated unaffected controls and dysmenorrhea samples into different blocks (R^2^X [1] = 0.373, R^2^X [2] = 0.148), respectively, suggesting altered gene expression profiles in the dysmenorrhea group. Although the PCA model provided an overview, the details of the differences underlying each cluster remained unclear. The supervised method, OPLS-DA, was then used to isolate the variables responsible for differences between control and dysmenorrhea samples on the first day of menstruation. The OPLS-DA score plots are shown in Figure 2C (R^2^X [1] = 0.180, R^2^X [2] = 0.304).

Potential markers, chosen on the basis of their contribution to the variation and correlation within the data set, were extracted from S-plots constructed following the OPLS analysis. The OPLS-DA loading S-plot, a plot of covariance versus correlation, in conjunction with the variable trend plots, allowed easier visualization of the data. The most significantly changed variables are plotted at the top or bottom of the S-plot, and those that do not vary significantly are represented in the middle. Genes showing significant differences in DM vs. NM (Figure 2D), were selected from the respective S-plots as potential markers of DM; these included *BMP3, IL6, IL8, IL1B, TNF, LEFTY2, BMP4, BMP6, NODAL, IL1F5, IL11, CSF1* and *GDF5.*

### Cytokine Gene qRT-PCR Array Analysis of PBMCs from Dysmenorrheic Women and Unaffected Controls on the Seventh day before the Menstrual Period (The Secretory Phase)

We analyzed cytokine gene expressions in PBMCs from dysmenorrheic women on the seventh day before the menstrual period and compared it to that of controls (Table 1). We found that 11 genes were differentially expressed (FC ≥2, *P*<0.05) with nine up-regulated and two down-regulated (Table 1). In samples from women with primary dysmenorrhea, the expression of some pro-inflammatory cytokines was markedly higher than in the controls.

**Table 1:** Genes revealed by quantitative RT-PCR analysis to be differentially expressed in women with primary dysmenorrhea on the seventh day before menstruation.

Gene	Normalized ΔCt		2∧-ΔCt		FC
	Nor	Dys	Nor	Dys	
*IL1A*	13.4±0.6	10.6±2.3*	9.4E-05	6.6E-04	7.1
*IL1B*	5.5±0.3	3.2±2.4*	2.2E-02	1.1E-01	4.9
*CSF2*	16.3±1.3	14.3±0.9*	1.3E-05	4.9E-05	3.8
*IL6*	10.4±0.6	8.7±2.4*	7.5E-04	2.4E-03	3.3
*IL21*	15.6±2.1	14.3±1.0	2.1E-05	4.8E-05	2.3
*TNF*	7.2±0.3	5.8±1.3*	6.9E-03	1.9E-02	2.7
*TNFRSF11B*	17.4±0.5	16.2±1.0	5.8E-06	1.3E-05	2.3
*IL5*	10.8±0.3	9.8±0.5*	5.7E-04	1.1E-03	2.0
*BMP3*	13.7±0.4	12.7±0.5*	7.4E-05	1.5E-04	2.1
					
*LEFTY2*	11.3±0.4	12.5±0.3**	4.0E-04	1.7E-04	−2.3
*BMP4*	13.7±0.9	15.0±0.2*	7.4E-05	3.0E-05	−2.5

Normalized ΔCt = Ct (GOI) – avg. (Ct (HKG)), where GOI is each gene of interest, and HKG are the housekeeping genes. Fold change (FC) = 2^−ΔΔCt = ^2^−ΔCt (Dys)^ ÷ 2^−ΔCt (Nor)^.

The greatest up-regulation in expression observed was in *IL-1A* and *IL-1B* (Table 1) (*P*<0.05). Other notable markedly up-regulated genes in the primary dysmenorrhea group include *CSF2*, *IL6*, *IL21*, *TNF*, *TNFRSF11B*, *IL5*, and *BMP3*. Genes down-regulated in samples from women with primary dysmenorrhea (Table 1) were *BMP4* (*P*<0.05) and *LEFTY2* (*P*<0.01) (Tab. 1).

To determine the biological meaning of the altered gene expression, genes where FC was ≥2 were uploaded to DAVID, and the resulting functional annotation chart inspected. DAVID-dependent analyses yielded 13 biological processes, molecular functions and pathways relating to the gene list (P-value cut-off, <0.05). The top biological processes or pathways associated with these genes were the Jak-STAT signaling pathway, the acute inflammatory response, and apoptosis (Table 2). Other top 10 GO terms and pathways are also listed in Table 2. These genes with altered expression patterns in the secretory phase of menstruation were also associated with endometrial decidualization; their functions and possible roles in decidualization are listed in Table 3.

**Table 2:** DAVID analysis of genes differentially expressed in women with primary dysmenorrhea on the seventh day before menstruation.

Category	Term or pathway	P Value	Genes	Regulation
KEGG_PATHWAY	Jak-STAT signaling pathway	7.2E-04	*CSF2, IL6, IL5, IL21*	Up
GOTERM_BP_FAT	Acute inflammatory response	1.1E-03	*IL6, IL1B, IL1A*	Up
KEGG_PATHWAY	Apoptosis	5.5E-03	*TNF, IL1B, IL1A*	Up
KEGG_PATHWAY	T cell receptor signaling pathway	9.4E-03	*CSF2, TNF, IL5*	Up
GOTERM_BP_FAT	Positive regulation of angiogenesis	1.6E-02	*IL1B, IL1A*	Up
GOTERM_BP_FAT	Negative regulation of hormone secretion	2.4E-02	*IL6, IL1B*	Up
GOTERM_BP_FAT	Leukocyte migration	4.0E-02	*TNF, IL1B*	Up
KEGG_PATHWAY	MAPK signaling pathway	5.2E-02	*TNF, IL1B, IL1A*	Up
GOTERM_BP_FAT	Positive regulation of cell differentiation	7.9E-02	*IL6, IL21*	Up
				
KEGG_PATHWAY	TGF-beta signaling pathway	1.6E-02	*BMP4, LEFTY2*	Down

**Table 3:** Functions and possible roles of genes differentially expressed in women with primary dysmenorrhea during the menstrual cycle.

Gene	Secretory phase(day -7)	Menstrual phase(day 1)	Repair phase(day 5)
***IL1A***	Promoted decidualization [51].		
***IL5***	Promoted eosinophil and mast cellmediated-inflammation. Involved intissue oedema before menstruationphase [43].		
***CSF2***	Induced by TNF/IL1B. Enhanced release of arachidonic acid (AA). Neutrophil chemo-attractant [39].		
***TNFRSF11B***	Pro-inflammatory cytokine.		
***TNF***	Regulated decidualization. Increased IGFBP-1 expression [19].	Increased PGs, endothelin and Ang production, MMPs expression and OT-induced Ca^2+^ transients [19], [24].	
***IL1B***	Positively (no exogenous cAMP) regulated decidualization. Stimulated production of prostaglandins (PGs) [40].	Stimulated PGs, OT, Ang, and endothelinproduction. Induced the expression ofMMPs [20], [22], [41], [52], [53], [54].	Induced the production of PGs, FGF, PDGF and VEGF in macrophages [55].
***IL6***	Increased secretion of PGs to promote decidualization indirectly [41].	IL-6 increased OT secretion in humanmyometrium, and *OTR* mRNA expression.Induced the expression of MMP11 [20], [23].	Induced PGs, FGF, PDGF and VEGF production [55].
***LEFTY2***	Inhibited decidualization [44].	Triggered menstrual ECM degradation via MMPs [9], [56].	Over-expression in wound tissue [56].
***BMP3***	Antagonized the activity of other BMPs [57].	Antagonized the activity of other BMPs [57].	Antagonized the activity of other BMPs [57].
***BMP4***	Involved in decidualization [58].	Inhibited inflammation following physiologicalinjury [30]. Inhibited the hypoxic inductionof COX-2 in smooth muscle cells [31].Increased heme oxygenase1 (HO-1) expressionand PPAR activity [33], [36]. Inhibited the transcriptionof estrogen receptor [59].	Involved in wound healing [60].
***IL21***	Pro-inflammatory cytokine in T-cellmediated inflammation [61].		Enhanced T-cell immunity [61].
***BMP6***		BMP6 increased HO-1 gene expression andactivity [34].	
***MSTN***		Low expression promotes fast musclecontraction [38].	
***NODAL***		Increased MMP-2 expression in glioma cell lines[47]. Activated caspase-3 and caspase-9 inepithelial ovarian cancer cells [62].	
***IL1F5***		Anti-inflammatory activity [63].	
***IL11***		A pleiotropic cytokine with anti-apoptotic, anti-inflammatory and hematopoietic potential[64], [65], [66].	
***GDF11***		Involved in the proliferation and differentiation of stem and progenitor cells [67].	
***GDF5***		Induced MMP-2 expression in periodontalligament cells [48].	Low expression in the proliferative phase of normal menstruation. Enhanced periodontal wound healing/regeneration [68].
***IL8***		Chemokine	Chemokine
***IL1F6***			Inflammatory repair [63].
***IL9***			Induced by IFNs and, IL-21. Enhanced T-cell and mast cell activity [69].
***IL13***			Inhibited T-cell immunity [70].
***IFNA2***			Increased expression of IGF1-R and EGF-R [71], [72].
***PDGFA***			Promoted endometrial stromal proliferation. Induced MMP11 expression [73].
***INHBA***			Inhibited pituitary FSH synthesis and secretion to reduce endometrial repair [74].
***TNFSF4***			Associated with inflammation [75].

### Cytokine Gene qRT-PCR Array Analysis of the PBMC from Unaffected Controls and Dysmenorrheic Women on the First Day of the Menstrual Period

On the first day of menstruation, the level of progesterone in these dysmenorrheic women was only 2.2±0.5 nM, but it was 44.3±14.0 nM on the seventh day before menstruation, indicating the withdrawal of progesterone. Plasma concentrations of progesterone (P4), 17b-estradiol (E2), follicle-stimulating hormone (FSH) and luteinizing hormone (LH) during menstrual cycle in primary dysmenorrheic women were shown in Table S1.

We analyzed cytokine gene expression in PBMCs on the first day of menstruation. In dysmenorrheic women 14 genes were differentially expressed (FC >2 or FC = 2, *P*<0.05) compared to controls, with nine up-regulated and five down-regulated (Table 4). DAVID analysis of these genes (P-value cut-off <0.05) found significant pathway associations, the most prominent being the TGF-beta and Toll-like receptor signaling pathways (Table 5).

**Table 4:** Quantitative RT-PCR array analysis of differentially expressed genes in women with primary dysmenorrhea on the first day of menstruation.

Gene	Normalized ΔCt		2∧-ΔCt		FC
	Nor	Dys	Nor	Dys	
***BMP4***	13.6±1.1	15.6±1.3**	8.3E-05	2.0E-05	−4.2
***BMP6***	5.1±1.8	6.2±0.8*	3.0E-02	1.3E-02	−2.3
***GDF5***	13.8±2.2	15.9±1.0*	7.0E-05	1.7E-05	−4.2
***GDF11***	9.7±4.2	11.9±0.5	2.6E-04	1.2E-03	−4.6
***LEFTY2***	11.5±0.1	12.8±0.6**	3.5E-04	1.4E-04	−2.5
***NODAL***	11.7±1.2	13.2±0.8**	2.9E-04	1.0E-04	−2.8
***IL1F5***	14.8±1.7	16.5±1.5*	3.6E-05	1.1E-05	−3.4
***IL11***	16.4±1.4	17.6±0.6*	1.2E-05	5.2E-06	−2.3
***MSTN***	14.4±1.7	15.7±1.1*	4.6E-05	1.9E-05	−2.4
***BMP3***	14.4±1.3	12.8±0.9*	4.6E-05	1.4E-04	3.1
***IL1B***	6.1±0.7	4.9±0.6*	1.4E-02	3.3E-02	2.3
***TNF***	7.7±0.5	6.7±0.4**	4.7E-03	9.8E-03	2.2
***IL6***	10.7±0.6	9.7±0.8	6.0E-04	1.2E-03	2.1
***IL8***	5.4±1.4	3.0±0.9**	2.3E-02	1.3E-01	5.4

**Table 5:** DAVID analysis of differentially expressed genes in women with primary dysmenorrhea on the first day of menstruation.

Category	Term or pathway	P Value	Genes	Regulation
GOTERM_MF_FAT	Growth factor activity	3.1E-13	*BMP4, NODAL, LEFTY2, GDF5, GDF11,* *MSTN, IL11, BMP6*	Down
KEGG_PATHWAY	TGF-beta signaling pathway	3.9E-07	*BMP4, NODAL, LEFTY2, GDF5, BMP6*	Down
GOTERM_BP_FAT	Regulation of cell proliferation	8.8E-03	*BMP4, NODAL, GDF11, IL11*	Down
GOTERM_BP_FAT	BMP signaling pathway	2.6E-02	*BMP4, BMP6*	Down
GOTERM_BP_FAT	Response to wounding	3.7E-02	*MSTN, IL11, BMP6, GDF5,*	Down
GOTERM_BP_FAT	Response to glucocorticoid stimulus	4.5E-02	*BMP4, MSTN*	Down
				
KEGG_PATHWAY	Toll-like receptor signaling pathway	5.2E-06	*IL6, TNF, IL8, IL1B*	Up
GOTERM_BP_FAT	Inflammatory response	2.8E-03	*IL6, IL8, IL1B*	Up
GOTERM_BP_FAT	Leukocyte migration	2.0E-02	*TNF, IL1B*	Up
KEGG_PATHWAY	Apoptosis	4.9E-02	*TNF, IL1B*	Up

**Figure 3:**
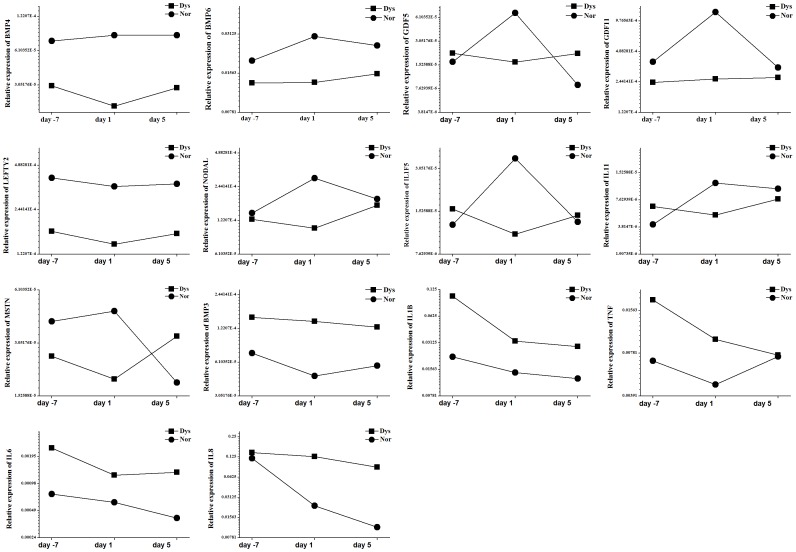
Expression of primary dysmenorrhea-related genes by quantitative RT-PCR array on the seventh day before menstruation, and the first and fifth days of menstruation. Compared with the unaffected control group, the primary dysmenorrhea group has the relatively low expression of genes (*BMP6*, *GDF5*, *GDF11*, *NODAL*, *IL1F5*, *IL11* and *MSTN*), and high expression of pro-inflammatory cytokines (*IL1B, TNF, IL6,* and *IL8*).

The gene with the most markedly reduced expression was *BMP4*. Other notably down-regulated TGF-β superfamily member genes included *BMP6*, *GDF5*, *GDF11*, *LEFTY2*, *NODAL,* and *MSTN*, which are associated with inhibition of excessive inflammatory responses and wound healing (Table 3). Besides the down-regulation of TGF-β superfamily members, expression of two anti-inflammatory cytokines (*ILF5* and *IL11*) was also down-regulated in primary dysmenorrhea. In the unaffected control group, we observed that the expression of several genes (*BMP6*, *GDF5*, *GDF11*, *NODAL*, *IL1F5*, *IL11* and *MSTN*) was clearly increased on the first day of menstruation, compared with the seventh day before and the fifth day of menstruation (Figure 3). However, the expression of these genes was very low in the primary dysmenorrhea group (Figure 3), suggesting that they are closely associated with the control of inflammation and pain in menstruation.

As the shown in the figure 3, gene expressions of several pro-inflammatory cytokines (*IL1B, TNF, IL6,* and *IL8*) were low in the unaffected control group during the whole menstrual cycle. However, they were highly expressed in the primary dysmenorrhea group.

**Figure 4:**
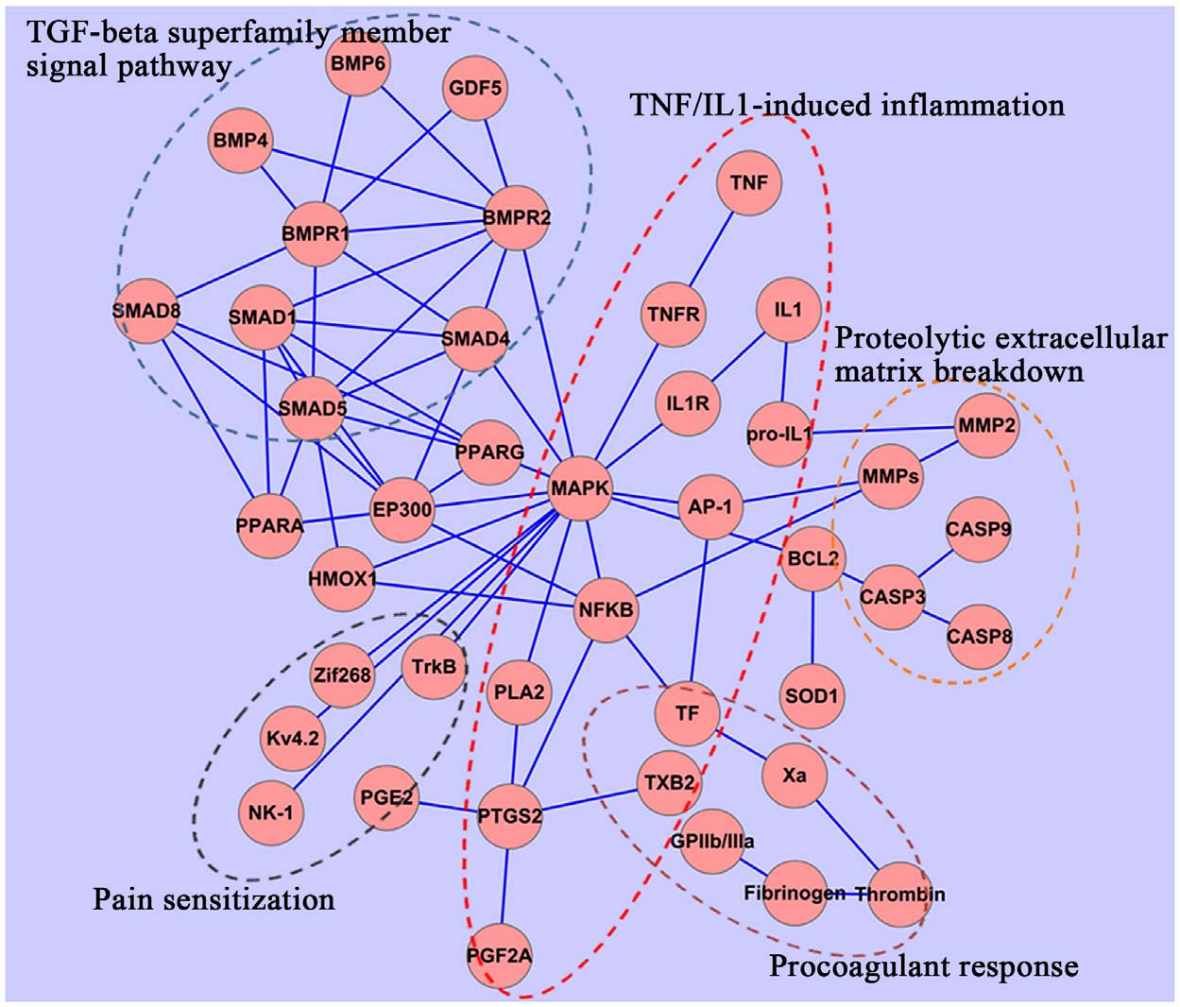
A simplified representation of biological cross-talk between multiple TNFα/IL-1-induced actions and the TGF-β superfamily member signaling pathway. TGF-β family members may interfere with the multiple roles of TNF-α/IL-1via HO-1, p300, PPARα, and PPARγ.

In order to identify further the roles of genes with altered expression profiles in women with primary dysmenorrhea, we investigated the biological association between these genes and chemicals (PGF2α and Oxytocin) which induce uterine hypercontractility and subsequent menstrual pain. Analysis using SciMiner showed that co-occurrence of pro-inflammatory cytokines and PGF2α or Oxytocin is frequent, suggesting that pro-inflammatory cytokines are potent inducers of the release of chemicals promoting uterine contraction. SciMiner analysis also revealed the co-occurrence of TGF-β superfamily members with TNF-α/IL-1. Their biological relationships were further extracted from the PubMed database using ALIBABA, and the resulting network of inter-connected objects visualized graphically using Cytoscape. Figure 4 illustrates that TGF-β family members could affect (inhibit) the multiple roles of TNF-α/IL-1via intermediary molecules, including HO-1, p300, PPARα, and PPARγ. In addition, pro-inflammatory cytokine and TGF-β superfamily members could affect the expression of MMPs.

**Figure 5:**
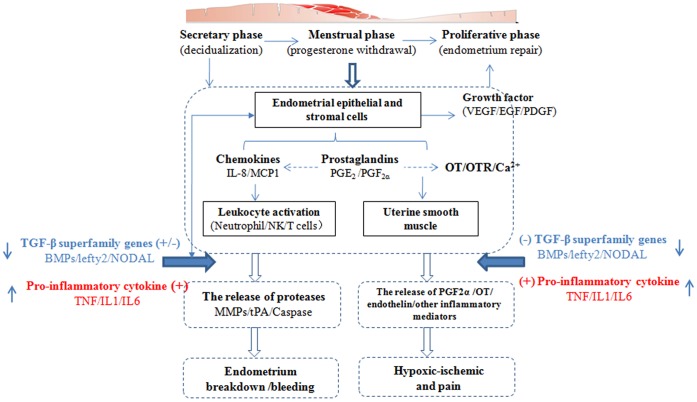
A model of the biological basis of the onset of menstrual pain. Menstruation is a response to the withdrawal of progesterone and depends on complex interactions between ovarian hormones and the immune system. A variety of immune factors not only regulate the inflammation and pain in menstruation, but also affect decidualization, tissue breakdown and early repair in the menstruation process. ↑, up-regulation of gene expression regulation; ↓, down-regulation of gene expression; (+), positive regulation; (−), negative regulation.

The functions and possible roles in menstrual pain of genes identified in our analyses are detailed in Table 3 and Figure 5.

### Cytokine Gene qRT-PCR Array Analysis of the PBMC from Dysmenorrheic Women and Unaffected Controls on the Fifth day of the Menstrual Period (The Late Perimenstrual Phase)

In the late perimenstrual, or early proliferative phase of menstruation, the human endometrium regenerates after shedding. We analyzed the cytokine gene expression in PBMCs from dysmenorrheic women on the fifth day of menstruation and compared them to those of unaffected females (Table 6). We found that 15 genes were differentially expressed (FC ≥2, *P*<0.05) with seven up-regulated and eight down-regulated. The potential functions of these genes and effects associated with menstruation are shown in Table 3. DAVID analysis of these genes (P-value cut-off of, <0.05) identified significantly associated biological processes and pathways. The top identified associated processes/pathways were positive regulation of cell proliferation (associated with down-regulated gene) and the inflammatory response (associated with up-regulated genes); the top 10 associated pathways are listed in Table 7.

**Table 6:** Quantitative RT-PCR array analysis of differentially expressed genes in women with primary dysmenorrhea on the fifth day of menstruation.

Gene	Normalized ΔCt		2∧-ΔCt		FC
	Nor	Dys	Nor	Dys	
***BMP4***	13.6±0.6	15.1±1.3*	8.3E-05	2.9E-05	−2.9
***LEFTY2***	11.4±0.5	12.5±0.5*	3.6E-04	1.7E-04	−2.1
***IFNA2***	14.2±1.5	15.6±0.8*	5.2E-05	2.0E-05	−2.6
***PDGFA***	9.1±0.1	10.0±0.5**	1.9E-03	9.5E-04	−2.0
***IL1F6***	10.1±0.5	11.2±0.9**	9.4E-04	4.2E-04	−2.3
***IL21***	13.4±0.2	14.7±0.7**	9.4E-05	3.8E-05	−2.5
***IL9***	14.2±0.6	15.3±1.4*	5.3E-05	2.5E-05	−2.2
***TNFSF4***	6.4±0.4	7.4±0.5**	1.2E-02	6.0E-03	−2.0
***IL1B***	6.3±0.2	5.1±1.5*	1.2E-02	2.8E-02	2.3
***IL6***	11.3±0.7	9.6±0.8*	4.0E-04	1.3E-03	3.2
***IL8***	6.5±1.9	3.5±2.1	1.1E-02	8.8E-02	7.8
***BMP3***	14.1±0.2	13.0±0.7*	5.7E-05	1.3E-04	2.2
***GDF5***	16.8±1.0	15.5±0.9*	8.5E-06	2.1E-05	2.5
***IL13***	14.3±0.4	13.1±0.3**	5.1E-05	1.1E-04	2.2
***INHBA***	11.4±0.7	10.2±0.4**	3.8E-04	8.3E-04	2.2

**Table 7:** DAVID analysis of differentially expressed genes in women with primary dysmenorrhea on the fifth day of menstruation.

Category	Term or pathway	P Value	Genes	Regulation
GOTERM_BP_FAT	Positive regulation of cell proliferation	2.8E-05	*BMP4, TNFSF4, PDGFA, IL9, IL21*	Down
GOTERM_MF_FAT	Growth factor activity	6.3E-05	*BMP4, PDGFA, LEFTY2, IL9*	Down
GOTERM_BP_FAT	Response to wounding	7.4E-05	*IFNA2, TNFSF4, PDGFA, IL9, IL1F6*	Down
GOTERM_BP_FAT	Inflammatory response	4.5E-04	*IFNA2, TNFSF4, IL9, IL1F6*	Down
GOTERM_BP_FAT	Response to estradiol stimulus	2.8E-02	*BMP4, PDGFA*	Down
GOTERM_BP_FAT	Angiogenesis	7.4E-02	*BMP4, PDGFA*	Down
				
GOTERM_BP_FAT	Inflammatory response	2.8E-03	*IL6, IL8, IL1B*	Up
GOTERM_BP_FAT	Response to wounding	8.0E-03	*IL6, IL8, IL1B*	Up
GOTERM_BP_FAT	Negative regulation of hormone secretion	1.2E-02	*IL6, IL1B*	Up
KEGG_PATHWAY	TGF-beta signaling pathway	7.8E-02	*INHBA, GDF5*	Up

Expression of *BMP4* and *PDGFA*, which are associated with tissue repair and angiogenesis, was significantly down-regulated on the fifth day of the menstrual period. Levels of several cytokines (*IFNA2*, *IL21*, *IL9*, *IL1F6*, and *TNFSF4*) related to endometrial cell proliferation were also reduced. In addition, the inflammatory cytokines (*IL1B*, *1L-6*, and *IL8*) were dysregulated, suggesting a lasting inflammatory response during the late perimenstrual phase in primary dysmenorrhea.

## Discussion

The peripheral immune response is involved in the pathogenesis, progression and prognosis of many tissue-specific diseases. Previous studies have established that local immune-inflammation in the endometrium plays a key role in the regulation of menstruation [16], [17], [18]. However, the relationship between peripheral immune function and primary dysmenorrhea is yet to be elucidated. In the present study, we investigated cytokine gene expression profiles of PBMCs derived from six females experiencing painful menstruation and three unaffected controls at three different stages of the menstrual cycle, with a view to identification of potential peripheral immunologic features related to the occurrence of primary dysmenorrhea. The results indicated that the main changes in gene expression of PBMCs in primary dysmenorrhea were up-regulation of those encoding pro-inflammatory cytokines and down-regulation of TGF-β superfamily transcripts. To the best of our knowledge, this is the first report of cytokine gene expression signatures in PBMCs from women with primary dysmenorrhea and offers new insights into the molecular pathogenesis of this disease.

In primary dysmenorrhea, the expression levels of pro-inflammatory cytokine genes (*IL1B, TNF, IL6* and *IL8*) were significantly increased on the first day of menstruation, whereas those of anti-inflammatory cytokines (*ILF5* and *IL11*) were markedly reduced compared to unaffected controls. Many studies have shown that pro-inflammatory cytokines could stimulate the synthesis or release of PGF2α and OT, which induce uterine hypercontractility, decrease endometrial blood flow, and cause pain. Both IL-1β and TNF-α treatment increased PGF2α production in cultured endometrial stromal cells, which was associated with augmentation of COX-2 protein expression [19], [20]. Experimentally infected mares expressed more *IL-6*, *IL-8*, *IL-1β*, and *TNF-α* mRNA in the cervical star region and produced high concentrations of PGE_2_ and PGF2α in allantoic fluid, leading to abortion or birth of a precociously mature foal [21]. Pro-inflammatory cytokines also increase oxytocin/Ca^2+^ signaling, which has important roles in myometrial contractions. For example, IL-1β increased OT secretion in human deciduas through the production of prostaglandins [22]. IL-6 promoted uterine *OTR* mRNA expression and binding capacity in human smooth muscle cells through tyrosine and serine phosphorylation pathways [23]. TNF-α increased OT-stimulated Ca^2+^ transients in human myometrial cells and this effect was abolished by progesterone [24]. In addition, pro-inflammatory cytokines (IL-1β, TNF-α and IL-6) may cause blood vessel constriction [25], [26], increase procoagulant activity [27] and induce the excitability of sensory neurons [28]. Although there is no proof that the gene changes in PBMCs could increase uterine contraction, the increased expression of pro-inflammatory cytokine genes may produce multiple actions contributing to primary dysmenorrhea.

In the present study, we found that the expression of TGF-β family genes (*BMP4*, *BMP6*, *GDF5*, *GDF11*, *LEFTY2*, *NODAL*, and *MSTN*) in primary dysmenorrhea was down-regulated on the first day of menstruation. The TGF-β system signals via protein kinase receptors and Smad mediators to regulate a plethora of biological processes, including immune regulation, wound healing, and inflammation [29]. In the *Drosophila melanogaster* model, BMP-4 has been shown to be an important inhibitor of inflammation following sterile injury [30]. BMP-4 could inhibit the hypoxic induction of COX-2 by a MAPK-independent pathway in human peripheral pulmonary artery smooth muscle cells [31]. Suppression of inflammatory mediator production by BMP4 may be through the Smad-associated mechanism acting on NF-κB [32]. This inhibition occurs by competition between Smad 1 and the NF-κB complex for P300, which is an essential transcriptional co-activator for both. Moreover, BMPs could induce the expression of heme oxygenase-1 (HO-1) [33], [34], which exhibits important anti-inflammatory properties through the MAPK pathway and cytoprotective action through inhibiting oxidative damage [35]. BMP-4 could also activate PPARα and PPARγ to suppress TNF-α actions [36]. BMP-4 was reported to prevent the development of thermal hyperalgesia and mechanical allodynia in rats, suggesting that it has analgesic activities [37]. In addition, *MSTN* is significantly down-regulated in primary dysmenorrheic women. Low expression of this gene has been associated with faster muscle contraction [38], suggesting that *MSTN* may be a marker for uterine hypercontractility in primary dysmenorrhea.

Our results clearly demonstrate that differential expression of PBMC cytokine genes between unaffected and dysmenorrheic women occurs not only in the menstruation phase, but also across the whole menstrual cycle. The role of the inflammatory response differs during the cyclical changes of the endometrium and is hormonally regulated. During the secretory phase, pro-inflammatory cytokines (IL-1β and TNF-α) are involved in endometrial decidualization. PGE_2_, stimulated by pro-inflammatory cytokines, significantly increased the decidualization via the cAMP pathway [19], [39], [40], [41]. Due to the presence of progesterone, pro-inflammatory cytokines did not cause an abnormally increased inflammatory response in the endometrium. Progesterone effectively inhibited the TNFα-induced release of PGF2α and OT, and markedly depressed the expression and activation of MMPs through NF-κB in endometrial tissue [3]. The interactions of pro-inflammatory cytokines and hormones cause endometrium differentiation in preparation for subsequent menstruation. During the perimenstrual phase, the withdrawal of progesterone eliminates its inhibition of the inflammatory response, and triggers a cascade of inflammatory mediators (TNF-α, PGF2α, MMPs, etc.), culminating in the breakdown of the endometrial extracellular matrix by cytokines, followed by menstrual bleeding. The inflammation resolves after menstruation, and a weak inflammatory response contributes to endometrial repair, partly via PGE_2_ [9], [10]. The local mechanisms of resolution of inflammation during the proliferative phase have yet to be delineated. A recent study showed that TNF-α induced more PGF2α from decidual cells after pretreatment with E2/P4 than from normal oviductal epithelial cells [42], suggesting that decidual tissue may be the main source of inflammatory mediators. Once the decidualized endometrium is expelled from the uterus, the strong inflammatory response may naturally transition to a weaker response.

The altered gene expression profiles of PBMC cytokines may not only induce excessive inflammation, but also affect the menstrual events (decidualization, proteolytic extracellular matrix breakdown) to exacerbate primary dysmenorrhea indirectly. In the secretory phase, progesterone induces differentiation of endometrial stromal cells (ESCs), into decidual cells. Decidualization is characterized histologically by the appearance of larger and rounder cells surrounding the spiral arteries and eventually spreading through most of the endometrium. Pro-inflammatory cytokines may positively regulate decidualization (no exogenous cAMP), such as IL-1β, TNFα, IL-6 and CSF2 [19], [40], [41]. IL-5 promotes the function of eosinophil and mast cells, which secrete many vasoactive, nociceptive, and pro-inflammatory molecules and correspond closely to endometrial oedema in the secretory phase [43]. The growth factor TGF-β family members also are involved in decidualization. LEFTY2 negatively regulates decidualization [44]. Together, the up-regulated pro-inflammatory and down-regulated TGF-β family member genes may facilitate decidualization. Since it has been shown that the decidual cells release more PGF2α after pretreatment with E2/P4 than normal epithelial cells without E2/P4 pretreatment, it is possible that excessive decidual transformation in the secretory phase leads to increased production of inflammatory substances, inducing pain in the menstrual phase.

Menstrual bleeding is secondary to ECM proteolysis induced by MMPs. MMPs have a pivotal role in ECM breakdown in the human endometrium and are modulated by TGF-β family members [45]. LEFTY-A triggers menstrual ECM degradation by up-regulating the expression of *MMP*s *3*,*7*, and *9* [9]. BMP-4 has been shown to induce the expression of *MMP1* and *9* [46], and NODAL and GDF5 are able to up-regulate expression of *MMP-2* [47], [48]. These reports suggest that the down-regulated expression of TGF-β genes may reduce their contribution to endometrial breakdown. Furthermore, the down-regulation of TGF-β family genes may be compensated for by up-regulation of pro-inflammatory genes, which can also induce MMPs, to ensure menstrual bleeding. However, pro-inflammatory cytokines may induce the excessive expression and activation of MMPs via NF-κB or AP-1, whereas TGF-β family members may regulate MMPs both positively and negatively (depending on cell type or the type of stimulation) [49]. Taken together, the disruption of the balance of TGF-β family and pro-inflammatory gene expression in the menstrual phase would be expected to affect endometrial breakdown.

Clear differences in PBMC gene expression between unaffected and dysmenorrheic women were also observedin the repair phase of the menstrual cycle. Gene annotations from DAVID demonstrated an up-regulated inflammatory response (*IL6*, *IL8*, *IL1B*), and down-regulation of cell proliferation (*BMP4*, *TNFSF4*, *PDGFA*, *IL9*, *IL21*) and response to wounding (*IFNA2*, *TNFSF4*, *PDGFA*, *IL9*, *IL1F6*). BMP-4 has been shown to activate angiogenesis via the induction of the expression of vascular endothelial growth factor (VEGF) [50]. These changes suggest that lasting acute inflammation, impaired T-cell immunity, and delayed endometrium repair occur after the experience of menstrual pain.

In summary, the gene expression pattern observed in young women with primary dysmenorrhea revealed dysregulated inflammation responses with extensive down-regulation of TGF-β family member genes related to anti-inflammatory responses, along with the up-regulation of genes coding for pro-inflammatory cytokines. The gene expression changes occurred not only on the first day of menstruation, but throughout the whole cycle, and may be involved in the regulation of menstrual events (e.g. decidualization, endometrium breakdown, and repair) and act indirectly to exacerbate primary dysmenorrhea.

## Supporting Information

Table S1 **Plasma concentrations of progesterone (P4), 17b-estradiol (E2), follicle-stimulating hormone (FSH) and luteinizing hormone (LH) on the seventh day before (−7d), and the first (1d) and the fifth (5d) days of menstruation in primary dysmenorrheic women.**
(DOC)
