# Supplementary material for: Altered Cytokine Gene Expression in Peripheral Blood Monocytes across the Menstrual Cycle in Primary Dysmenorrhea: A Case-Control Study
Source: PLoS One. 2013 Feb 4;8(2):e55200. doi: 10.1371/journal.pone.0055200 (PMC3563666; doi:10.1371/journal.pone.0055200)
Supplement: Table S1 — Plasma concentrations of progesterone (P4), 17b-estradiol (E2), follicle-stimulating hormone (FSH) and luteinizing hormone (LH) on the seventh day before (−7d), and the first (1d) and the fifth (5d) days of menstruation in primary dysmenorrheic women. (DOC) [file pone.0055200.s001.doc]

Table S1. Plasma concentrations of progesterone (P4), 17b-estradiol (E2), follicle-stimulating hormone (FSH) and luteinizing hormone (LH) on the seventh day before (-7d), and the first (1d) and the fifth (5d) days of menstruation in primary dysmenorrheic women.

| Group | **P4** | **E2** | **FSH** | **LH** |
| --- | --- | --- | --- | --- |
| **-7d**  **1d**  **5d** | 44.3±14.0  2.2± 0.5**  2.2±0.6 | 783.1±234.3  123.3±29.1**  175.4±40.5 | 2.3±0.4  6.5±1.4**  7.1±`1.5 | 5.6±3.3  6.0±2.2  5.4±0.9 |

Values represent the mean ± SD (n = 6). ***P* < 0.01 indicated a significantly different from -7d groups (Student’s t-test).
